# Live Cell FRET Imaging Reveals Amyloid β-Peptide Oligomerization in Hippocampal Neurons

**DOI:** 10.3390/ijms22094530

**Published:** 2021-04-26

**Authors:** Yang Gao, Stefan Wennmalm, Bengt Winblad, Sophia Schedin-Weiss, Lars O. Tjernberg

**Affiliations:** 1 Department of Neurobiology, Care Sciences and Society, Division of Neurogeriatrics, Karolinska Institutet, 171 64 Solna, Sweden; yang.gao@ki.se (Y.G.); bengt.winblad@ki.se (B.W.); sophia.schedin.weiss@ki.se (S.S.-W.); 2 SciLifeLab, Department of Applied Physics, Biophysics, Royal Institute of Technology, 171 65 Solna, Sweden; stewen@kth.se; 3 Theme Inflammation and Aging, Karolinska University Hospital, 141 86 Huddinge, Sweden

**Keywords:** amyloid β-peptide, oligomerization, aggregation, FRET

## Abstract

Amyloid β-peptide (Aβ) oligomerization is believed to contribute to the neuronal dysfunction in Alzheimer disease (AD). Despite decades of research, many details of Aβ oligomerization in neurons still need to be revealed. Förster resonance energy transfer (FRET) is a simple but effective way to study molecular interactions. Here, we used a confocal microscope with a sensitive Airyscan detector for FRET detection. By live cell FRET imaging, we detected Aβ42 oligomerization in primary neurons. The neurons were incubated with fluorescently labeled Aβ42 in the cell culture medium for 24 h. Aβ42 were internalized and oligomerized in the lysosomes/late endosomes in a concentration-dependent manner. Both the cellular uptake and intracellular oligomerization of Aβ42 were significantly higher than for Aβ40. These findings provide a better understanding of Aβ42 oligomerization in neurons.

## 1. Introduction

Alzheimer disease (AD) is the most common cause of dementia, and affects mainly older people [1]. It is projected to affect more than 100 million people worldwide in 2050, and its pathogenesis remains unclear [2]. The aggregation of the amyloid β-peptide (Aβ) is believed to play a crucial role in the pathogenesis of AD [3].

Aβ is a 38–43 amino acid peptide cleaved from a transmembrane protein, the Aβ precursor protein (APP) [4]. It is generated intracellularly and secreted to the extracellular space [5,6]. Among the various Aβ isoforms, Aβ42 is more toxic than other species because it is more prone to aggregate [7]. Aβ oligomers—both intracellular and extracellular—are suggested to impair synaptic function and contribute to cognitive decline during the early stages of AD [8]. Increased intracellular Aβ42 has been shown to correlate with AD neuropathology [9,10]. However, more studies on intracellular Aβ oligomers are needed to understand the pathogenesis in early AD.

Oligomeric Aβ exist in a dynamic equilibrium, which is sensitive and influenced by the environment, and biophysical analysis can affect the oligomers [11,12]. Several techniques have been used for studying Aβ oligomerization, such as Thioflavin T assays, SDS polyacrylamide gel electrophoresis (PAGE), and fluorescence microscopy [13]. However, only a few of them are suitable to use in living cells under physiological conditions.

Förster resonance energy transfer (FRET) is widely used to measure protein interactions in molecular biology. Due to its strong dependence on the distance between the donor and acceptor [14], it has been used for studying cellular Aβ oligomerization and showed unique capability [15].

Several studies have shown that extracellular Aβ could be internalized and form aggregates in cells [15,16]. However, some key questions regarding the fate of internalized Aβ still need to be addressed, such as under what conditions, in which subcellular compartments, and to what extent Aβ could oligomerize in neurons.

In this study, we performed live cell FRET imaging to detect Aβ42 oligomerization in primary neurons, by using a high-resolution confocal microscope with a sensitive Airyscan detector. We found that monomeric Aβ could be taken up into primary neurons and oligomerize mainly in the lysosomes and/or late endosomes in a concentration-dependent manner.

## 2. Results

### 2.1. Internalized Aβ Oligomerizes in Lysosomes/Late Endosomes

**Figure 1:**
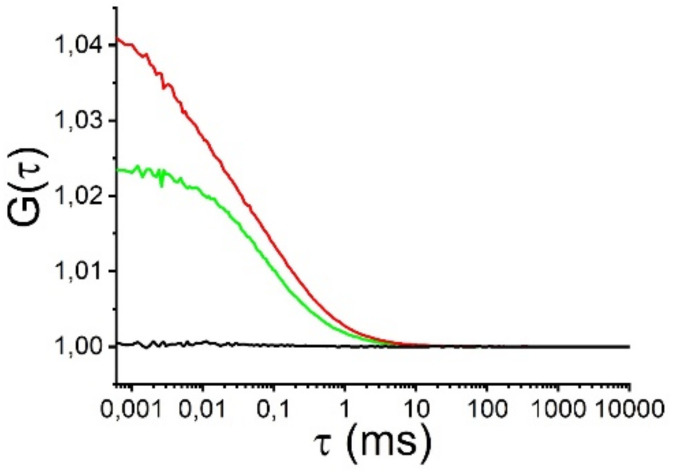
Fluorescence cross-correlation spectroscopy (FCCS) measurements of a sample consisting of 500 nM HF488 Aβ42 (green) + 500 nM HF647 Aβ42 (red). Auto- (green and red) and cross-correlation (black) curves were collected in 10-minute measurements after 24 h incubation. Laser power: 0.4% 488 nm and 1.2% 633 nm. The lack of cross-correlation indicates that a very low number of oligomers, if any, were present. If there were, the black cross-correlation curves would have an amplitude rising above the 1.0-line, like the green and red auto-correlation curve.

Fluorescence correlation spectroscopy (FCS), fluorescence cross-correlation spectroscopy (FCCS), and the combination of FRET and FCS (FRET-FCS) were used to investigate the oligomerization state of Aβ in the cell culture medium. These techniques have been shown capable of detecting Aβ oligomers in solution and FRET-FCS can even detect FRET-active oligomers at fractions below 1% compared to monomers [17]. We prepared 500 nM HiLyte™ Fluor 488 (HF488) Aβ42 and 500 nM HiLyte™ Fluor 647 (HF647) Aβ42 in the cell culture medium. The FCCS analysis showed that Aβ aggregation was low in the medium after 24 h (Figure 1, Figures S1–S3).

**Figure 2:**
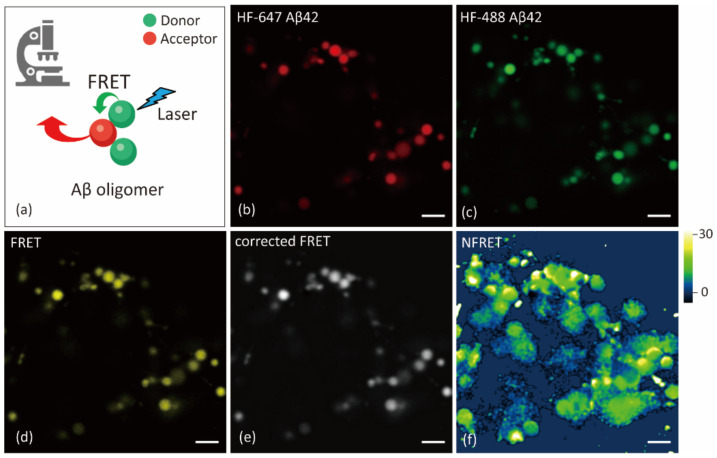
Förster resonance energy transfer (FRET) analysis to detect aggregated Aβ42 in primary neurons. (**a**) Principle of FRET. Equimolar Aβ42 monomers labeled with HF488 and HF647 are used for oligomerization detection. If the emission of excited donor overlaps with the absorption of the acceptor, energy transfers from donor to ground state acceptor when their distance is 10 nm or less. (**b**–**d**) Acceptor channel (640 nm excitation, 650–700 nm emission), donor channel (488 nm excitation, 480–520 nm emission) and FRET channel (488 nm excitation, 650–700 nm emission) of representative FRET image from confocal microscope. (**e**–**f**) The corrected FRET and normalized FRET (NFRET) image from ImageJ PixFRET plug-in. Scale bar: 2 μm.

Sensitized emission FRET was used to detect Aβ oligomerization in primary neurons (Figure 2a). In this approach, we excited the donor HF488 Aβ42, which transfers energy directly to the acceptor HF647 Aβ42 if it is within 10 nm distance, resulting in emission from the acceptor. The emitted light is then detected by a confocal microscope using an emission filter for the acceptor. Theoretically, it can be excluded that FRET arises due to the low mean monomer-to-monomer distance of Aβ even at high concentration. Thus, the FRET signal in this study indicates Aβ oligomerization.

It should be noted that fluorescence crosstalk from donor, cross-excited acceptor, and noises such as autofluorescence may interfere with the analysis. To acquire the corrected FRET from crosstalk, spectral bleed-through parameters of the donor and acceptor were determined with cells treated with only HF488 Aβ42 or only HF647 Aβ42, respectively. The cell autofluorescence was measured in a non-treated control group. The image analysis was processed using the ImageJ plug-in PixFRET.

Primary hippocampal neurons (21 days in vitro) were incubated in the presence of 500 nM HF488 Aβ42 and/or 500 nM HF647 Aβ42 for 24 h and imaged by a confocal microscope equipped with an Airyscan detector. Both HF488 Aβ42 and HF647 Aβ42 were accumulated in the vesicles of the soma (Figure 2b–c). After correction, a FRET signal was found in most of the Aβ-containing vesicles (Figure 2d–e).

The corrected FRET can still be affected by the concentration of fluorophores. To get normalized FRET, the corrected FRET value was divided by the square root of the product of donor and acceptor intensities in each pixel. Figure 2f shows the normalized FRET of the vesicles in the soma of neuron.

**Figure 3:**
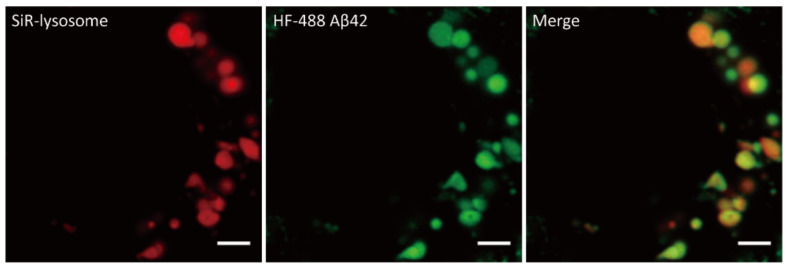
Internalized Aβ42 in primary neurons. DIV 21 neurons were treated with 1000 nM HF488 Aβ42 (green) for 24 h. SiR-lysosome (red) was used to label lysosome and late endosomes. Scale bar: 2 μm.

Next, to identify the subcellular location where Aβ is accumulated, we used the lysosomal marker SiR-lysosome in neurons that were treated with 1000 nM Aβ42 for 24 h. Most of Aβ-containing vesicles were colocalized with the lysosomal marker (Figure 3 and Figure S4). This suggests that Aβ42 was internalized and oligomerized into lysosomes and late endosomes.

### 2.2. Aβ42 Oligomerization Occurs at Both Surface and Vesicles of Neurons

**Figure 4:**
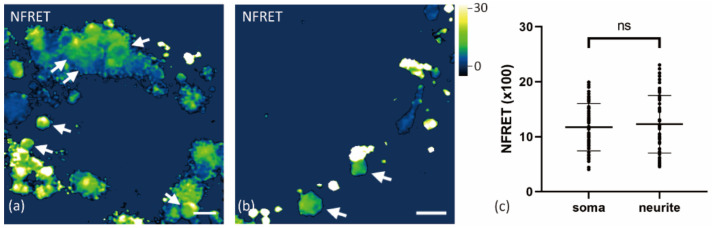
Comparison of FRET between Aβ vesicles in soma and neurites. Primary neurons were treated with 500 nM HF488 Aβ42 and 500 nM HF647 Aβ42 for 24 h. (**a**) Normalized FRET (NFRET) image of Aβ vesicles (white arrow) in soma. (**b**) NFRET image of Aβ vesicles (white arrow) in neurites. (**c**) NFRET values of 63 vesicles (1 cell per image from 8 images) in soma and 64 vesicles (16 images) in neurite were compared using the Mann–Whitney test (*p* > 0.05). Data are mean ± S.D. Scale bar: 2 μm.

**Figure 5:**
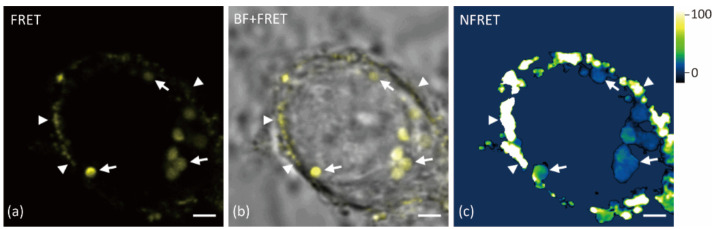
FRET images of Aβ on the cell surface. Primary neurons were treated with 500 nM HF488 Aβ42 + 500 nM HF647 Aβ42 for 24 h. (**a**) FRET channel; (**b**) merged image of brightfield (BF) and FRET channel; (**c**) normalized FRET image. The white arrow indicates the Aβ vesicles in soma, and the arrowhead indicates the Aβ on the cell surface. Scale bar: 2 μm.

A comparison of Aβ oligomerization in vesicles distributed in soma with vesicles in neurites showed no significant differences (*p* > 0.05, Figure 4). The weak Aβ signal on the cell surface was difficult to distinguish from the background while imaging the vesicles with high intensities in soma without oversaturation. Thus, higher laser power and detector gain were applied to measure the Aβ aggregation on the cell surface. The normalized FRET value of Aβ42 on the neuron surface or nearabout could be several times higher than in vesicles of soma and neurites (Figure 5). This is consistent with the group treated with lower Aβ concentrations (Figure S5). Similar strong oligomerization was also observed along the neurites, although we did not identify if it came from the surface of neurites.

### 2.3. Internalized Aβ42 Oligomerization in Soma Is Concentration-Dependent

**Figure 6:**
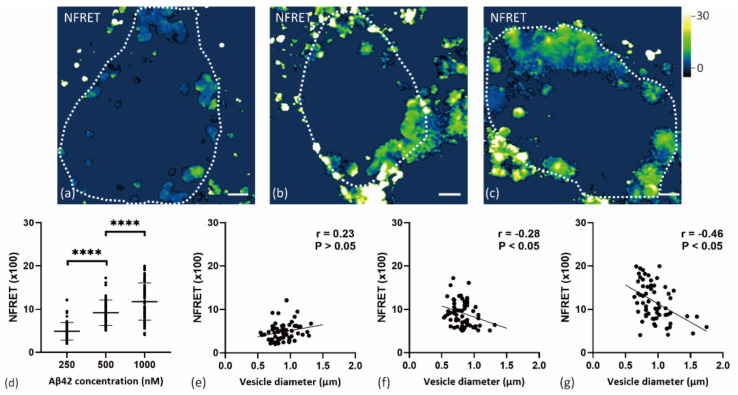
Internalized Aβ42 oligomerization in neuronal soma. (**a**–**c**) Normalized FRET (NFRET) image of vesicles in soma. Primary neurons were treated with 250 nM Aβ42, (**a**) or 500 nM Aβ42, (**b**) or 1000 nM Aβ42 (**c**) for 24 h. (**d**) NFRET values of 63 vesicles (1 cell per image from on the average 8 images) from each group were calculated and compared using the ANOVA test (*p* < 0.0001). Data are mean ± S.D.; (**e**–**g**) Pearson correlation of Aβ vesicle size and NFRET values in neurons treated with 250 nM Aβ42, (**e**) or 500 nM Aβ42, (**f**) or 1000 nM Aβ42 (**g**) for 24 h. Scale bar: 2 μm. ****, indicates *p* < 0.0001.

To further confirm the association between Aβ42 oligomerization and concentration in soma, primary neurons were treated with three different Aβ concentrations (HF488 Aβ42 and HF647 Aβ42 at 1:1 ratio; 250, 500, and 1000 nM Aβ in total) for 24 h. As shown in Figure 6, Aβ oligomerization occurred in all the three groups, and increased with extracellular Aβ42 concentration. We also found a negative correlation between the diameter of the Aβ vesicles and the normalized FRET level in two cell groups treated with higher Aβ concentration. The smaller vesicles tended to present higher oligomerization levels in cells treated with 1000 nM Aβ42 (r = −0.46, *p* < 0.05) and 500 nM Aβ42 (r = −0.28, *p* < 0.05). There was no significant correlation in the 250 nM group (r = 0.23, *p* > 0.05).

### 2.4. Internalized Aβ42 Oligomerizes in Neurons With Higher Efficiency Than Aβ40

Finally, we treated primary neurons with fluorescently labeled Aβ40, and compared the polymerization of internalized Aβ40 and Aβ42. Confocal image analysis showed that more Aβ42 were taken up into neurons than Aβ40 using the same concentrations for 24 h, and the Aβ42 oligomerization level was significantly higher as well (Figure S6).

**Figure 7:**
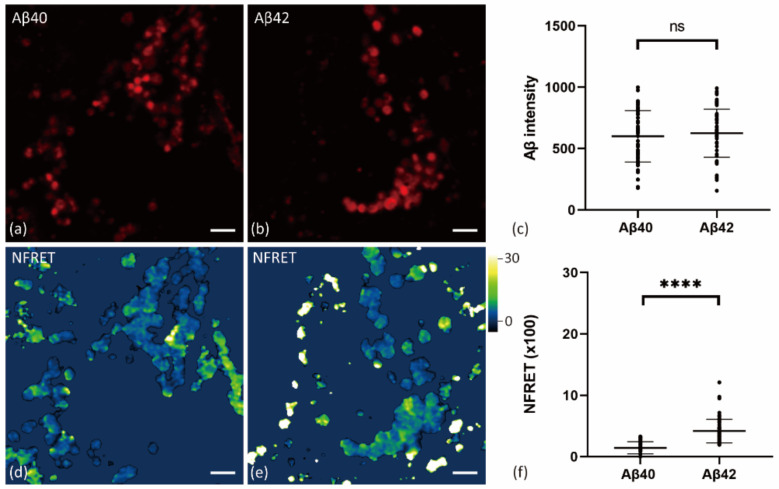
FRET images of neurons treated with Aβ40 or Aβ42. Primary neurons were treated with 1000 nM Aβ40 (**a**,**d**) or 250 nM Aβ42 (**b**,**e**) for 24 h (HF488 Aβ42 and HF647 Aβ42 at 1:1 ratio). (**a**–**b**) Confocal images of Aβ vesicles (HF647) in primary neurons; the same acquisition and display settings were used for both groups. (**c**) Aβ intensity (HF647) of selected vesicles in neurons treated with 1000 nM Aβ40 (*n* = 54, 1 cell per image from 8 images) and 250 nM Aβ42 (*n* = 65, 1 cell per image from 9 images) were compared using the Mann–Whitney test (*p* > 0.05). (**d**–**e**) Normalized FRET (NFRET) images of Aβ vesicles in primary neurons. (**f**) NFRET values of selected Aβ vesicles in neuron treated with Aβ40 and Aβ42 were compared using the Mann–Whitney test (*p* < 0.0001). Data are mean ± S.D. Scale bar: 2 μm. ****, indicates *p* < 0.0001. “ns” indicates *p* > 0.05.

Because the difference of oligomerization could be due to different Aβ concentrations in vesicles, we then compared vesicles with similar intensity of Aβ42 and Aβ40. Aβ concentration in vesicles of cells treated with 1000 nM Aβ40 for 24 h was comparable to some of those treated with 250 nM Aβ42 (Figure 7a,b). Therefore, we selected vesicles with similar Aβ concentrations from these two groups and compared their normalized FRET level. Aβ42 treatment was found to induce more oligomerization than Aβ40, under similar Aβ concentrations in vesicles (Figure 7c–f).

**Figure 8:**
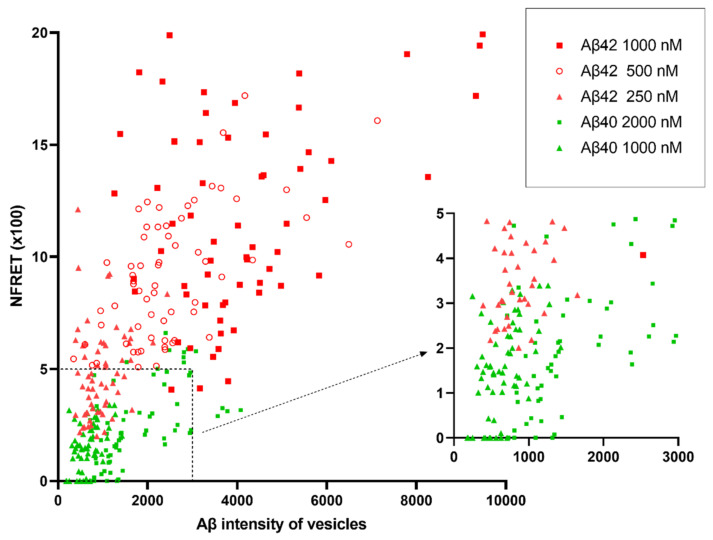
Scatter plot of normalized FRET (NFRET) and Aβ intensity (HF647) in vesicles of neuronal soma. Primary neurons were treated with different concentrations of Aβ40 (green) or Aβ42 (red) for 24 h (HF488 Aβ and HF647 Aβ at 1:1 ratio). The right graph shows enlarged details in the dashed box.

Figure 8 shows the scatter plot of normalized FRET values and Aβ concentration in the vesicles of cells treated with different Aβ concentrations. The aggregation of both Aβ42 and Aβ40 showed strong trends to increase with their concentrations in vesicles. Despite lower external concentration, Aβ42 still aggregated to a higher extent than Aβ40.

## 3. Discussion

Aβ aggregation is thought to have a crucial role in the progression of AD. However, the detection of Aβ oligomers is challenging due to it lacking stability and being easily influenced during analysis [18]. FRET has been used to measure molecular distances at the nanoscale for a long time. Here, by measuring FRET with a confocal microscope with an Airyscan unit, we confirmed that extracellular monomeric Aβ could be internalized into the murine hippocampal neurons and oligomerized in lysosomes and/or late endosomes. The high normalized FRET signal on the cell surfaces indicated part of the Aβ oligomers might have already formed before getting into lysosomes/late endosomes.

It has been suggested that intraneuronal Aβ accumulation could be an early event in the pathogenesis of AD [4]. In this study, extracellular Aβ is taken up by neurons and oligomerized intracellularly, making it a possible source of intraneuronal Aβ aggregation. We observed strong Aβ oligomerization occurred in primary neurons treated with only 250 nM Aβ42 for 24 h. Previously, Hu et al. did not detect Aβ aggregation in SHSY5Y cells treated with 500 nM Aβ42 or below for five days, using agarose gel electrophoresis-SDS [16]. This might be due to the cellular uptake difference of Aβ, or because Aβ oligomers are SDS-instable species and this analysis could result in disassembly of oligomers [13].

In our study, Aβ in neurons aggregated in a concentration-dependent manner. This is consistent with previous studies in SHSY5Y cells and in buffers [16,19]. Besides, the size of Aβ vesicles also showed a significant negative correlation with aggregation in cells with 24 h treatment of higher Aβ concentration, but not in lower concentration. We suggest that cells with lower Aβ concentration treatment could be at an earlier stage of uptake at 24 h, compared with those with high concentrations.

The Aβ oligomer distribution in neurons is also interesting. It is reported that more lysosomes are located in soma than in neurites [20]. However, the oligomerization level in lysosomes/late endosomes of soma and neurites seems similar. We also identified the strongest normalized FRET signal on the neuron surface based on the following consideration. First, the fluorescence on the cell surface lacked movement during imaging, different from those in the cellular organelles. Secondly, such distinctly high normalized FRET was observed only on the cell border and along neurites, but not in soma. Besides, it has been reported that Aβ forms oligomers on lipid rafts in the cell membrane [21].

Regarding intraneuronal Aβ42 aggregation, another question is where this progress begins. First, we detected only little Aβ42 oligomerization at a 1000 nM concentration in the cell culture medium. Secondly, we noticed high Aβ oligomerization on the surface of neurons. The values of normalized FRET were several times higher than in the soma. The normalized FRET could be affected by the distance-dependent FRET efficiency and the ratio of “FRET” donor-acceptors to total donors and acceptors [22]. In this study, such a large difference in the normalized FRET between soma and membrane cannot be explained by the FRET efficiency. Thus, we infer that lysosomes/late endosomes in soma contained non-FRET monomeric Aβ42. The concentration of Aβ42 in those vesicles is much higher than in extracellular space [16]. Low pH in the lysosomes/late endosomes might promote Aβ aggregation [19,23]. Considering the high concentration and low pH, Aβ monomers in these vesicles are more likely to be taken up extracellularly rather than depolymerized from oligomers. We suggest that at least part of Aβ is internalized in the monomeric form, then aggregates in the late endosomes/lysosomes. However, it is possible that part of Aβ oligomerizes at the cell surface before entering late endosomes/lysosomes. It is also possible that oligomers that formed in neurons are released and then reinternalized to the endo-lysosomal system.

Compared with Aβ42, Aβ40 is less prone to form fibrils [24]. In the present study, Aβ42 aggregation dominance in neurons was partly owing to the cellular uptake preference, which resulted in high Aβ42 concentration in vesicles. Under similar concentrations in vesicles, Aβ42 still oligomerized more than Aβ40, however the difference is less than we expected. Although normally Aβ42 is present at lower concentrations than Aβ40 [25], it can induce more oligomers due to the enhanced cellular uptake and its strong aggregation tendency. This novel finding is in line with our previous finding showing that elevated levels of Aβ42 in neurons is a risk in brains of those with AD [9]. Thus, we hypothesize that Aβ42 accumulation in neurons by enhanced uptake is an early pathogenic event in AD, and that inhibition of this process could be a pharmacological strategy for treating AD.

In this study, we chose sensitized emission FRET for several reasons. It is simple, nondestructive, suitable for live imaging, and our study also fitted the stoichiometry of 1:1 donor and acceptor. The fast and sensitive Airyscan unit allowed us to detect FRET signals in moving vesicles, but it also detected unwanted autofluorescence. To minimize the caused false positive FRET, autofluorescence was measured from the non-treated vesicles that present the fluorescence. However, detected autofluorescence existed only in part of the vesicles, so background subtraction from the whole image might underestimate the FRET of those compartments with less autofluorescence, or even result in false negative values. In addition, although the bleed-through parameters were determined carefully, it was still difficult to measure accurate FRET data quantitatively with sensitized emission FRET, especially when the FRET signal was weak. This limits the use of sensitized emission FRET in some applications, such as determining the lowest Aβ concentration or the earliest timepoint to aggregate. Nevertheless, live cell FRET imaging can provide in situ detection with almost no intervention, making it a robust and unique technique for oligomerization measurement, when designed properly and conducted under controlled conditions.

In summary, our study suggests that the Aβ42 monomer can be internalized in neurons and oligomerized in the late endosome/lysosomes with high efficiency. In such a way, Aβ42 oligomers might accumulate gradually in neurons over decades of a lifetime, and cause the neuronal dysfunction.

## 4. Materials and Methods

### 4.1. Synthetic Human Aβ Peptide and Preparation

Aβ (1–40) and Aβ (1–42) peptides, labeled with HiLyte™ Fluor 488 or HiLyte™ Fluor 647 at the N-termini, were purchased from AnaSpec (BioNordika, Solna, Sweden). Aβ peptides were reconstituted by adding 40–50 µl 1% NH_4_OH to 0.1 mg dry peptide and diluted to 1 mg/ml with PBS, then aliquoted and stored at −20 °C.

### 4.2. Primary Neuron Culture

Hippocampal neurons were isolated from the hippocampi of E16.5 C57/BL6 mice embryo brains, seeded on the inner 10 mm microwell of poly-d-lysine-coated glass bottoms of P35G-1.5-10-C culture dishes (MatTek Corporation), as described previously [26]. Cortical neurons were seeded at the pre-coated edges of the plate as a support layer. Both hippocampal and cortical neurons were grown in selective Neurobasal medium containing 2% B27 (Invitrogen) and 1% l-glutamine (Invitrogen) at 37 °C in a cell incubator (humidified, 5% CO_2_), and cultured for 21 days in vitro (DIV).

### 4.3. Lysosomes/Late Endosomes Labeling in Live Cell Imaging

Lysosomes/late endosomes were labeled using SiR-Lysosome (Spirochrome AG). Primary neurons were treated with 1000 nM SiR-Lysosome at 37 °C, 5% CO2, 0.5 h before imaging.

### 4.4. Confocal Microscope and FRET Measurements

Confocal and FRET images were acquired using a Zeiss LSM 800 confocal microscope with Airyscan detector, using a Plan-Apochromat 63×/1.4 oil-immersion objective and the 488 and 640 nm laser lines as excitation source, unless specified otherwise. For FRET measurements, the laser power and detector gain for all channels were fixed through the whole experiment. Each channel was subsequently scanned by a 32-channel gallium arsenide phosphide photomultiplier tube (GaAsP-PMT) Airyscan detector. The acquired FRET images were 452 × 452 pixels (scan zoom: 5.0×) with a scan speed of 2.2 μs/pixel (pixel size: 0.043 μm). The pinhole for the fluorescence channel was 5.0 AU; for the brightfield channel it was 1.0 AU. Five to ten images were captured per cell culture dish.

The cells were incubated with either HF488 Aβ or HF647 Aβ, or an equimolar mixture of them. The donor control group was imaged sequentially in donor emission (488 nm excitation, 490–580 nm emission) and donor bleed-through (488 nm excitation, 650–700 nm emission). The acceptor control group included acceptor emission (640 nm excitation, 650–700 nm emission) and acceptor crosstalk (488 nm excitation, 650–700 nm emission). The FRET group contained three image sets: donor emission, acceptor emission, and FRET channel (488 nm excitation, 650–700 nm emission). The non-treated neurons group that was used to detect the cell autofluorescence and background shared the same settings as the FRET group.

FRET analysis was performed using the ImageJ plug-in PixFRET as previously described [27]. Concisely, spectral bleed-through was first determined in each image acquired from the donor control group and acceptor control group, by using a constant model in PixFRET. A Gaussian blur value of 2.0 was applied during the determination. By applying PixFRET computation, the original FRET images were then corrected from the spectral bleed-through and generated the FRET and normalized FRET image. “FRET/sqrt (Donor*Acceptor)” was selected as the output of normalized FRET, in which values for each pixel were divided by the square root of donor and acceptor intensities from same pixels. Gaussian blur (2.0) and threshold (1.0) values were set for all the images. To clearly distinguish the low and high values, normalized FRET images were displayed in a lookup table “Green Fire Blue”. For comparison, the negative normalized FRET values were set to 0. The quantification of normalized FRET values and Aβ intensity in vesicles were measured with ImageJ. Aβ intensity was calculated with HF647 Aβ because acceptor emission is not affected by FRET. The sizes of Aβ vesicles were determined in the fluorescence channel; the vesicles were selected using the oval tool and the diameter was calculated from the measured area of vesicles by ImageJ (“Analyze-Measure”).

FCS, FCCS and FCS-FRET measurements were recorded using a Zeiss 780 confocal laser scanning microscope equipped for FCS, with a Zeiss water immersion objective, C-Apochromat 40 ×/1.2 NA. The measurements have been described previously [17]. In this study, FRET-FCS was performed on the cell-free neuronal culture medium with 24 h treatment of 500 nM HF488 Aβ42 and 500 nM HF647 Aβ42. The diffusion time (τ) refers to the mean time of fluorescent particles passing through the detection focus.

### 4.5. Statistics

Statistical tests were performed using the GraphPad Prism 8 software. Normality was assessed using D’Agostino and Pearson tests. The non-parametric Mann–Whitney test was used for the data that were not normally distributed. One-way ANOVA was used for comparison of three groups. Correlation analysis was tested by the Pearson method. When the *p* value was less than 0.0001, it is stated as “****”, and “*” for *p* < 0.05. If the p was greater than 0.05, it is stated as *p* > 0.05 or “ns”. The data were obtained from two primary culture preparations. For statistical analysis, 408 vesicles from at least 56 images/cells taken from ten culture dishes derived from two primary neuron preparations were analyzed.

## Data Availability

The data presented in this study are available on request from the corresponding author.

## Supplementary Materials

The following are available online at https://www.mdpi.com/article/10.3390/ijms22094530/s1.
