# Supplementary material for: Live Cell FRET Imaging Reveals Amyloid β-Peptide Oligomerization in Hippocampal Neurons
Source: Int J Mol Sci. 2021 Apr 26;22(9):4530. doi: 10.3390/ijms22094530 (PMC8123703; doi:10.3390/ijms22094530)
Supplement: Supplementary file 1 [file ijms-22-04530-s001.zip › ijms-1164906-supplementary.pdf]

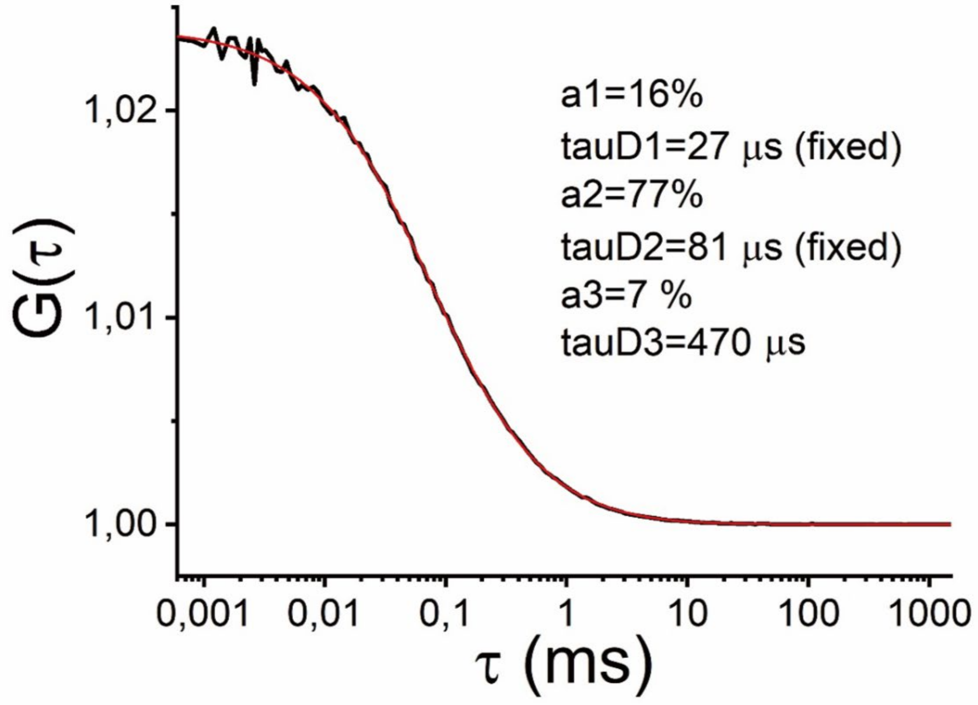

Figure S1. FCS measurement of Aβ42 oligomers. FCS curve recorded in the green channel on a sample consisting of 500 nM HF488 Aβ42 + 500 nM HF647 Aβ42. The two shortest diffusion times were fixed to tauD1=27 μs and tauD2=81 μs corresponding to free dye and HF488 Aβ42 monomers respectively, because the diffusion coefficient of HF488 Aβ42 is about three times slower than that of the free dye HiLyte488 (Wennmalm et al, 2015). The fit yielded that 16% of the amplitude belonged to the free dye, 77% to the Aβ42 monomer, and 7 % to a species of tauD3=470 μs. The slowest component indicates that a few oligomers of Aβ42 are present.

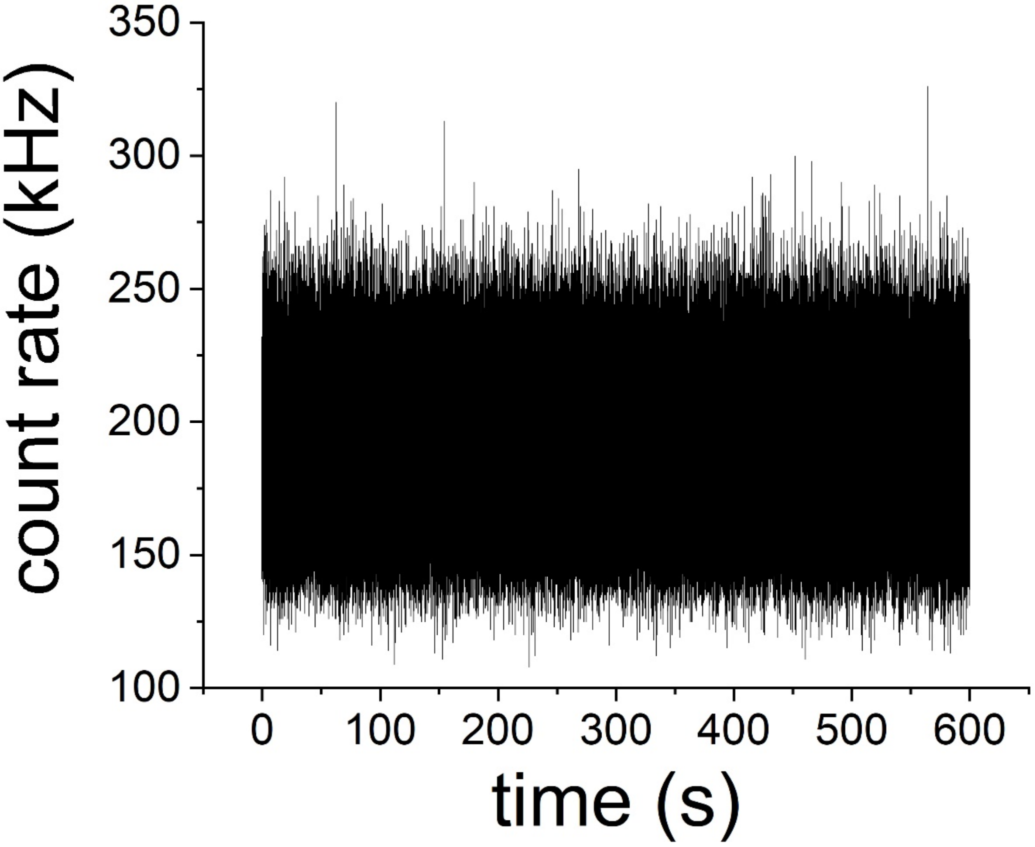

Figure S2. Intensity trace from a 10 min measurement on the same sample as in Figure S1. The lack of spikes in the intensity trace indicates that no large aggregates of Aβ42 are present.

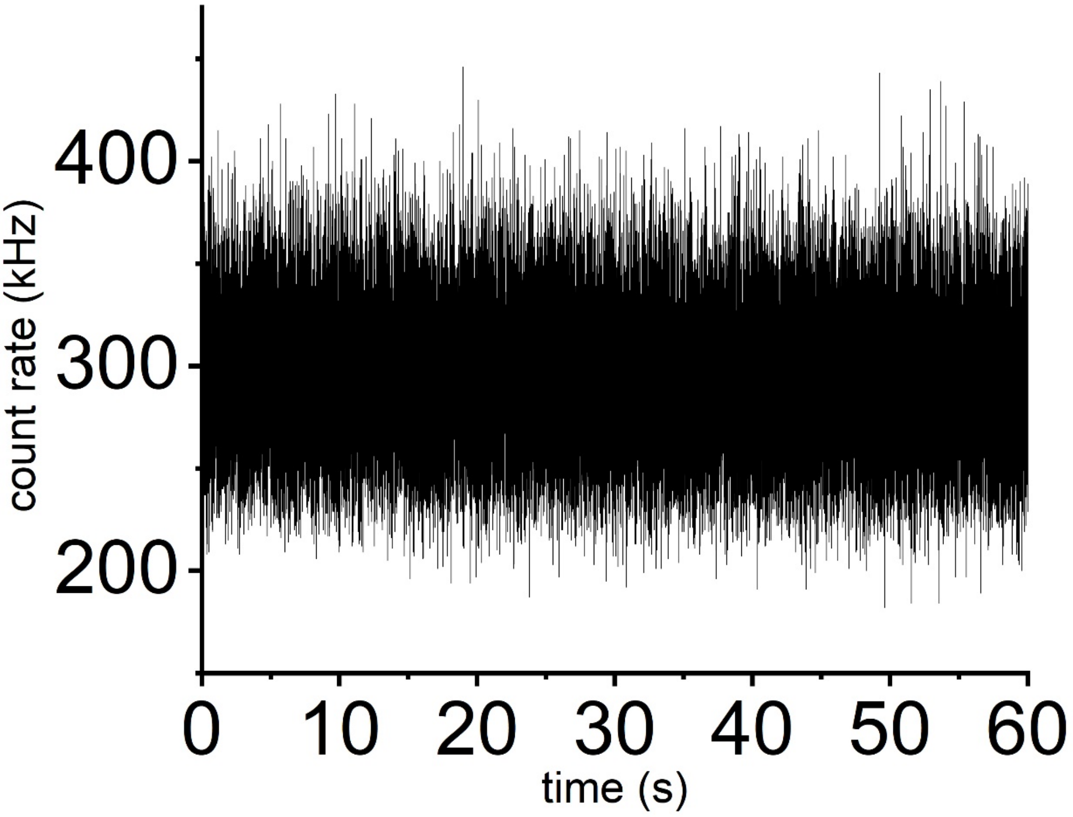

Figure S3. Intensity trace from a 60 s measurement on the same sample as in Figure S1. The lack of spikes in the intensity trace indicates that no large aggregates of Aβ42 are present.

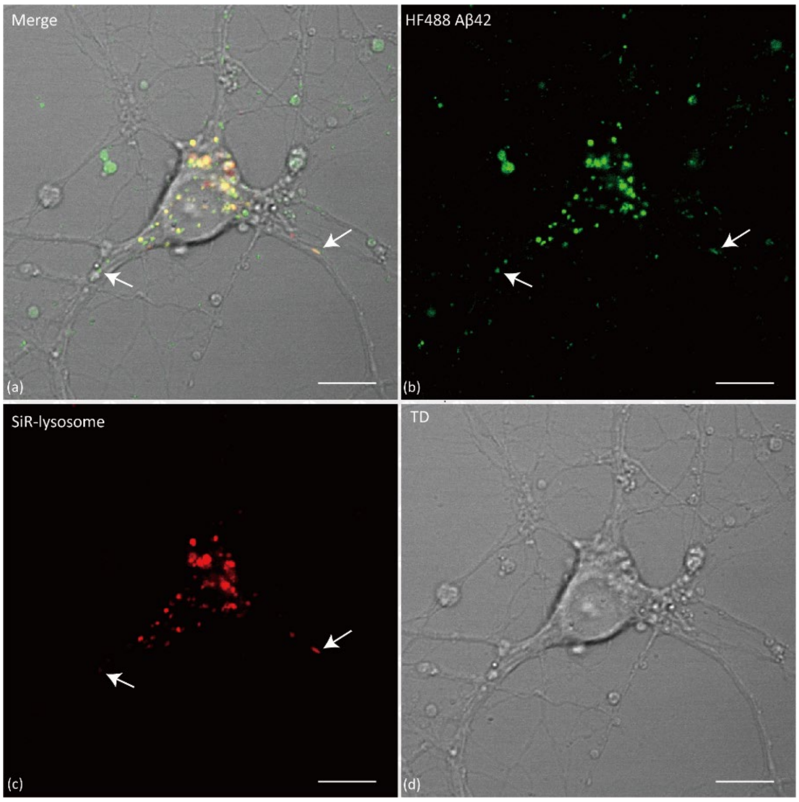

Figure S4. Confocal image of primary neurons (DIV21) treated with 1000 nM HF488 Aβ42 for 24 hours. Most of Aβ42 (green) were internalized into SiR-lysosome (red) positive vesicles in soma and a few Aβ42 vesicles were in neurites (white arrow). After 24 hours treatment with Aβ, the morphology of neurons is not altered. (**a**) Merged channel; (**b**) HF488 Aβ42; (**c**) SiR-lysosome; (d) Transmitted channel (TD). Scale bar: 10 μm. The images were acquired by Nikon A1 Plus confocal with Plan Apo VC 60x Oil Objective (NA = 1.4). Pinhole size: 26.82 μm. Pixel size: 0.11 μm. HF-488 Aβ42 channel, excitation: 488.0 nm, emission: 525.0 nm, GaAsP detector; SiR-lysosome channel, excitation: 640.8 nm, emission: 700.0 nm, PMT detector.

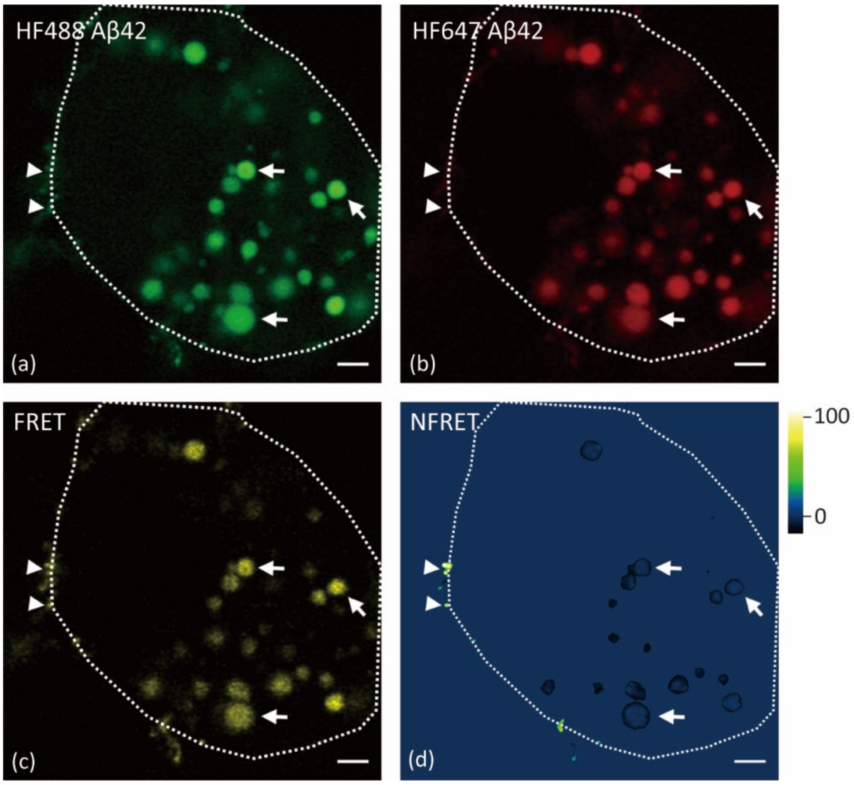

Figure S5. FRET images of Aβ on the cell surface. Primary neurons were treated with 125 nM HF488 Aβ42 + 125 nM HF647 Aβ42 for 24h. (**a**) HF488 Aβ42 (Gamma = 0.45); (**b**) HF647 Aβ42 (Gamma = 0.45); (**c**) FRET channel (Gamma = 1.0); (**d**) Normalized FRET (NFRET) image. The white arrow indicates the Aβ vesicles in soma, and the arrowhead indicates the Aβ on the cell surface. The cell body of neuron is outlined with the white dashed lines. Scale bar: 2 μm.

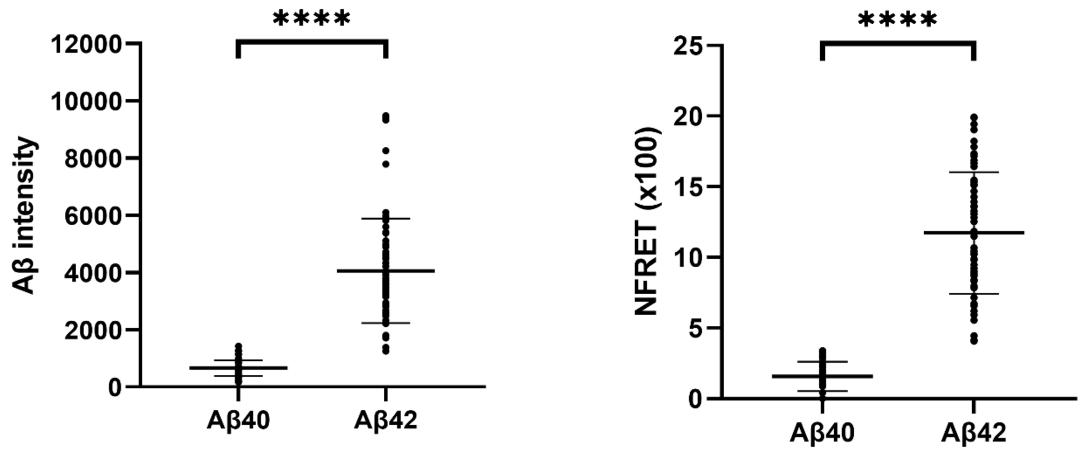

Figure S6. Comparison of primary neurons treated with 1000 nM Aβ40 or Aβ42 for 24h. Aβ intensity (HF647, left) and NFRET values (right) of Aβ vesicles in neurons treated with Aβ40 (N=60, 1 cell per image from 8 images) and Aβ42 (N=63, 1 cell per image from 8 images) were compared using Mann-Whitney test ($P < 0.0001$). Data are mean ± S.D.
